# How Problem-Solving Attitudes Link Catastrophic Thinking to Environmental Awareness Among Egyptian University Students: A Structural Equation Modeling Approach

**DOI:** 10.3390/ejihpe16020024

**Published:** 2026-02-12

**Authors:** Fatimah Ali Alhuraybi, Bassam M. A. Makram, Mohamed Sayed Abdellatif, Ashraf Ragab Ibrahim, Mohamed Ali Nemt-allah

**Affiliations:** 1 Department of Psychology, College of Education in Al-Kharj, Prince Sattam Bin Abdulaziz University, Al-Kharj 11942, Saudi Arabia; fa.alhuraybi@psau.edu.sa (F.A.A.); m.heby@psau.edu.sa (M.S.A.); 2 Department of Physics, College of Sciences and Humanities, Prince Sattam Bin Abdulaziz University, Wadi Al-Dawaser 11991, Saudi Arabia; 3 Educational Psychology and Statistics Department, Faculty of Education, Al-Azhar University, Cairo 35822, Egypt; ashrafibrahem.26@azhar.edu.eg (A.R.I.); mohamednamatallah.2026@azhar.edu.eg (M.A.N.-a.)

**Keywords:** catastrophic thinking, cognitive distortions, environmental awareness, problem-solving attitudes, university students, structural equation modeling

## Abstract

This study examined the mediating role of problem-solving attitudes in the relationship between catastrophic thinking and environmental awareness among university students using structural equation modeling. Two samples of undergraduate students from Al-Azhar University, Egypt, participated: a psychometric validation sample (N = 670) and a main study sample (N = 989). Participants completed three validated instruments assessing catastrophic thinking, problem-solving attitudes, and environmental awareness. Results revealed that catastrophic thinking was significantly negatively associated with environmental awareness both directly (β = −0.266) and indirectly through problem-solving attitudes (β = −0.172), with the indirect pathway accounting for approximately 39% of the total effect. The structural model demonstrated excellent fit to the data, and all hypothesized relationships were statistically significant. These findings suggest that catastrophic cognitions are associated with reduced environmental awareness both directly and through their negative relationship with problem-solving orientations that facilitate engagement with complex issues including environmental challenges. The study highlights the importance of addressing trait-level cognitive distortions alongside environmental content in education programs, as general catastrophic thinking patterns may impair environmental awareness even among students without climate-specific anxiety.

## 1. Introduction

Catastrophic thinking represents a trait-level cognitive distortion characterized by the automatic prediction of worst-case scenarios across life domains, exaggeration of negative event likelihood, and underestimation of coping abilities (Petrini & Arendt-Nielsen, 2020). This general pattern of repetitive negative thinking amplifies anxiety and depressive disorders by fostering overestimation of risks while minimizing resilience across health, social, and cognitive domains (Özdemir & Kuru, 2023; Petrini & Arendt-Nielsen, 2020). Individuals with catastrophic thinking tendencies systematically interpret ambiguous situations through a lens of impending disaster, whether in social relationships, physical health, or academic performance (Barchielli et al., 2022). This pervasive cognitive style has implications beyond personal well-being, potentially influencing how individuals engage with broader societal challenges including environmental issues.

Environmental awareness encompasses knowledge, attitudes, and concern regarding environmental issues, representing a multidimensional construct essential for sustainability (Arshad et al., 2020; Liobikienė & Poškus, 2019; Orbanić & Kovač, 2021). While conceptually distinct from actual behavior (Stern, 2000), environmental awareness serves as a critical cognitive–affective foundation for pro-environmental action. This concept includes understanding local and global environmental problems such as climate change, pollution, and biodiversity loss, alongside developing feelings of concern, empathy, and moral responsibility toward nature and future generations (Ienna et al., 2022; Luque-Alcaraz et al., 2024; Maurer et al., 2020; Woo & Kang, 2020). In the current global context, environmental awareness is critically important as humanity faces deepening ecological crises that threaten planetary habitability and human well-being (Kapecki, 2020; Isenaj et al., 2025). It serves as the psychological and cultural foundation for sustainable development, enabling informed decision-making, fostering pro-environmental citizenship, and supporting the behavioral changes essential for achieving the UN Sustainable Development Goals (Alharbi et al., 2025; Glavič, 2020; O’Flaherty & Liddy, 2017; Sonetti et al., 2019; Srisathan et al., 2024).

The relationship between general catastrophic thinking and environmental awareness represents an underexplored yet theoretically important connection. Individuals with catastrophic thinking tendencies exhibit compromised cognitive resources, heightened threat perception, and reduced capacity for constructive engagement across multiple life domains (Innocenti et al., 2023; Wullenkord & Reese, 2021). These general cognitive patterns may extend to environmental issues, where the tendency to catastrophize could either heighten concern about environmental problems or, alternatively, trigger defensive responses including avoidance and disengagement when environmental threats feel overwhelming (Janmaimool & Chudech, 2020; Verplanken et al., 2020). Understanding whether trait-level catastrophic thinking undermines or enhances environmental awareness has important implications for environmental education, as interventions may need to address general cognitive distortions that extend beyond domain-specific environmental concerns.

Problem-solving attitudes represent affective-cognitive orientations encompassing individuals’ confidence, approach tendencies, and personal control beliefs that fundamentally shape how they engage with challenges (Ozturk & Guven, 2016; Sturm & Bohndick, 2021). These attitudes comprise several core dimensions: self-efficacy or the belief in one’s capability to successfully tackle difficult problems (Hendriana et al., 2018; Evans et al., 2020), approach versus avoidance orientation that determines whether problems are viewed as meaningful opportunities or threats (Martino, 2019; Ozturk & Guven, 2016), and persistence in pursuing solutions despite obstacles (Ozturk & Guven, 2016). Beyond serving as mere psychological dispositions, problem-solving attitudes function as critical cognitive resources that regulate how individuals translate awareness into effective action (Schoenfeld, 1982). They guide attention allocation, strategy selection, and effort management during problem-solving processes (Martino, 2019; Ozturk & Guven, 2016), while also mediating stress responses and supporting adaptive coping mechanisms (Padmanabhanunni & Pretorius, 2023). Consequently, positive problem-solving attitudes enable individuals to implement their knowledge and strategies more effectively, particularly under challenging circumstances.

University students represent a strategically critical population for examining the interplay between catastrophic thinking, problem-solving attitudes, and environmental awareness. This demographic is experiencing a pivotal developmental period characterized by heightened uncertainty, stress, and identity formation that shapes enduring cognitive patterns including worry, rumination, and problem-solving styles (Barba-Sánchez et al., 2022; Brown et al., 2024; Diaz et al., 2023; Ibáñez et al., 2020; H. Liu et al., 2025; Peters & D’Penna, 2020). Furthermore, university students face uncertain futures in rapidly changing workplaces while simultaneously confronting existential environmental challenges, making them particularly vulnerable to climate anxiety, hopelessness, and future-related distress (Güler & Günday, 2024; Kennedy et al., 2025; Vandaele & Stålhammar, 2022; Wu et al., 2022). As future professionals, decision-makers, and community leaders, their current environmental awareness and problem-solving orientations will substantially influence policy development, organizational practices, and societal responses to environmental crises (Del Brío González et al., 2022; X. Liu et al., 2018; Morales-Baños et al., 2023; Navia et al., 2024; Pimdee, 2020; Saha et al., 2025).

Despite growing research on cognitive factors influencing pro-environmental attitudes and behavior, a critical gap persists in understanding how general catastrophic thinking—a trait-level cognitive distortion—influences environmental awareness through cognitive mediating pathways. Current literature has not examined whether individuals with catastrophic thinking tendencies show associations with reduced environmental awareness due to compromised cognitive resources and problem-solving orientations. While mediation models exist in environmental psychology, they typically position attitudes between perceived responsibility and green behavior (Zheng et al., 2020) or emotional intelligence between intentions and actions (Aziz et al., 2021), neglecting catastrophic cognitions and problem-solving orientations as intermediary mechanisms. Moreover, problem-solving skills are investigated predominantly within sustainability education contexts (Almulla & Al-Rahmi, 2023; Risopoulos-Pichler et al., 2020) without examining their role in transforming distressing ecological thoughts into constructive environmental engagement. Existing studies remain largely cross-sectional (Janmaimool & Khajohnmanee, 2019; Vieira et al., 2023), lacking integrative frameworks that simultaneously assess maladaptive climate-related cognitions, problem-solving attitudes, and environmental awareness within unified analytical models.

General catastrophic thinking tendencies may be negatively associated with environmental awareness through multiple pathways. First, catastrophic cognitions consume cognitive resources through rumination and worry, leaving less capacity for attention to and processing of environmental information (Pihkala, 2020; Schwartz et al., 2022). Second, catastrophic thinking may be negatively related to problem-solving attitudes—the confidence, persistence, and approach orientations that enable individuals to engage constructively with complex challenges (Zheng et al., 2020). Problem-solving attitudes represent critical cognitive resources that determine whether individuals engage with or avoid difficult issues across domains (Liobikienė & Poškus, 2019; Kousar et al., 2022). When catastrophic thinking is associated with lower problem-solving orientations, individuals may disengage from environmental issues that require sustained attention, critical thinking, and belief in the efficacy of solutions. Conversely, strong problem-solving attitudes may buffer against catastrophic thinking by promoting approach rather than avoidance orientations, thereby sustaining engagement with environmental awareness even among individuals prone to catastrophizing (Kesenheimer & Greitemeyer, 2021; Aziz et al., 2021).

The present study addresses this critical gap by examining the mediating role of problem-solving attitudes in the relationship between general catastrophic thinking and environmental awareness among university students. Using structural equation modeling, this research investigates whether trait-level catastrophic thinking is directly associated with reduced environmental awareness and whether this relationship operates indirectly through its negative association with problem-solving attitudes. Additionally, the study tests whether problem-solving attitudes positively predict environmental awareness, thereby serving as a crucial psychological mechanism that either amplifies or attenuates the influence of catastrophic cognitions on environmental concern. By integrating general catastrophic thinking tendencies and problem-solving orientations within a unified analytical framework, this study provides essential insights for understanding how pervasive cognitive distortions influence domain-specific awareness and for designing interventions that address general cognitive patterns while promoting environmental engagement among future professionals and decision-makers.

## 2. Materials and Methods

### 2.1. Research Design

This study employed a cross-sectional correlational design using structural equation modeling to examine the mediating role of problem-solving attitudes in the relationship between catastrophic thinking and environmental awareness among university students. Data were collected at a single time point through self-report questionnaires administered electronically.

### 2.2. Participants

The study involved two distinct samples: a psychometric validation sample and a main study sample. Participants were recruited via convenience sampling through classroom announcements and university email invitations. Inclusion criteria included current undergraduate enrollment, age 18–22 years, and Arabic language proficiency. Both samples comprised undergraduate students from Al-Azhar University, Egypt, specifically from the Faculty of Education and the Faculty of Humanities Studies, both located in Dakahlia Governorate. The psychometric sample (N = 670) ranged in age from 18 to 22 years (M = 19.40, SD = 1.09), while the main sample (N = 989) also ranged from 18 to 22 years (M = 20.46, SD = 1.69). A subsample of 108 participants from the psychometric validation sample completed the Catastrophic Thinking Scale a second time after a two-week interval to assess test–retest reliability. Detailed demographic characteristics of both samples are presented in Table 1.

### 2.3. Instruments

*The Catastrophic Thinking Scale* (CTS) was developed to assess catastrophic cognition among university students. Item generation followed a systematic process: (1) reviewing existing catastrophic thinking literature and clinical measures of catastrophizing (Crombez et al., 2020; Darnall et al., 2017; Laraki et al., 2023; Petrini & Arendt-Nielsen, 2020; Pike et al., 2021; Sullivan et al., 1995), (2) conducting focus groups with 15 university students to identify common catastrophic thoughts in social, physical, and cognitive domains, (3) generating an initial pool of 28 items, (4) expert review by three clinical psychologists who rated item relevance and clarity, and (5) pilot testing with 50 students followed by item refinement based on statistical analysis and participant feedback, resulting in the final 17-item scale.

The scale was originally constructed in Arabic, the participants’ native language, and subsequently translated into English through a rigorous process involving three independent expert translators who performed forward and backward translations to ensure conceptual equivalence. The final version comprises 17 items distributed across three dimensions: Social Concerns (SC) (6 items), Physical Concerns (PC) (6 items), and Cognitive Concerns (CC) (5 items). Respondents rate each item on a 5-point Likert scale ranging from 1 (Never) to 5 (Always), with higher scores indicating greater levels of catastrophic thinking (Appendix A). Confirmatory factor analysis conducted on the psychometric sample demonstrated acceptable model fit indices: χ^2^/df = 3.672, RMSEA = 0.063, GFI = 0.927, AGFI = 0.904, CFI = 0.936, NFI = 0.914, and TLI = 0.925. The three-factor structure exhibited strong convergent validity with composite reliability (CR = 0.864) and average variance extracted (AVE = 0.681) exceeding recommended thresholds. Internal consistency reliability coefficients for the subscales were excellent: SC (ω = 0.843, α = 0.841), Physical Concerns (ω = 0.837, α = 0.835), and Cognitive Concerns (ω = 0.841, α = 0.840). The total scale demonstrated high internal consistency (ω = 0.909, α = 0.909). Test–retest reliability assessed over a two-week interval with a subsample of 108 participants yielded substantial stability coefficients: SC (r = 0.704, *p* < 0.001), Physical Concerns (r = 0.767, *p* < 0.001), Cognitive Concerns (r = 0.587, *p* < 0.001), and total scale (r = 0.766, *p* < 0.001).

*The Attitudes Toward Problem Solving Scale* (ATPSS; Zakaria et al., 2004) was adapted for this study through translation from English to Arabic by three expert translators, followed by back-translation to ensure linguistic and semantic equivalence. The scale consists of 22 items distributed across three dimensions: Willingness to Engage (WE) (8 items), Problem-Solving Perseverance (PP) (6 items), and Self-Confidence (SFC) (8 items). Participants respond using a 5-point Likert scale ranging from 1 (Strongly Disagree) to 5 (Strongly Agree), with higher scores reflecting more positive problem-solving attitudes. Confirmatory factor analysis on the psychometric sample revealed excellent model fit: χ^2^/df = 1.037, RMSEA = 0.044, GFI = 0.972, AGFI = 0.966, CFI = 0.999, NFI = 0.980, and TLI = 0.999, substantially exceeding conventional adequacy thresholds. All three dimensions demonstrated strong internal consistency: WE (ω = 0.931, α = 0.931), PP (ω = 0.923, α = 0.923), and SFC (ω = 0.940, α = 0.940). The total scale exhibited high internal consistency (ω = 0.944, α = 0.945), indicating highly consistent measurement across all dimensions.

*The Environmental Awareness Questionnaire* developed by Al-Dosari and Abdellatif (2024) eliminates the need for translation. This 15-item instrument assesses three key dimensions of environmental awareness: Environmental Pollution (EP) (5 items), Environmental Balance (EB) (5 items), and Environmental Resources (ER) (5 items). Respondents indicate their agreement with each statement using a 5-point Likert scale from 1 (Strongly Disagree) to 5 (Strongly Agree), with higher scores denoting greater environmental awareness (Appendix B). Confirmatory factor analysis performed on the psychometric sample demonstrated superior model fit indices: χ^2^/df = 1.124, RMSEA = 0.014, GFI = 0.981, AGFI = 0.974, CFI = 0.999, NFI = 0.988, and TLI = 0.998, indicating excellent correspondence between the theoretical structure and observed data. The three subscales exhibited strong internal consistency: Environmental Pollution (ω = 0.900, α = 0.904), Environmental Balance (ω = 0.910, α = 0.911), and Environmental Resources (ω = 0.914, α = 0.914). The overall scale demonstrated excellent reliability (ω = 0.920, α = 0.920), confirming the instrument’s psychometric soundness for the target population.

### 2.4. Procedure

Data collection was conducted during the first semester of the 2025–2026 academic year, specifically from 28 September 2025 to 14 October 2025. All instruments were administered electronically through Google Forms, allowing for efficient and standardized data collection across both university campuses. Participants were informed about the study’s purpose, assured of confidentiality and anonymity, and provided informed consent before completing the questionnaires. The survey took approximately 20–25 min to complete. Participation was voluntary with no compensation offered, and students could withdraw at any point without consequence.

### 2.5. Data Analysis

Data were analyzed using SPSS version 27 for descriptive statistics, correlation analyses, and preliminary data screening, while structural equation modeling was conducted using AMOS version 26 to test the hypothesized mediation model. The analysis examined direct, indirect, and total effects, with bootstrapping procedures (5000 bootstrap samples) employed to generate bias-corrected 95% confidence intervals for mediation effects.

### 2.6. Hypotheses

Based on the theoretical framework and literature reviewed, this study tested the following hypotheses:

**H1.**  *Catastrophic thinking is negatively associated with problem-solving attitudes among university students.*

**H2.**  *Catastrophic thinking is negatively associated with environmental awareness among university students.*

**H3.**  *Problem-solving attitudes are positively associated with environmental awareness among university students.*

**H4.**  *Problem-solving attitudes mediate the relationship between catastrophic thinking and environmental awareness, such that catastrophic thinking is indirectly associated with environmental awareness through its negative relationship with problem-solving attitudes.*

## 3. Results

Before testing the hypothesized relationships, preliminary analyses were conducted to examine the correlational structure among study variables and assess potential common method bias. The correlation matrix presented in Table 2 reveals significant relationships among all study variables at the *p* < 0.001 level. Catastrophic thinking demonstrated significant negative correlations with problem-solving attitudes (r = −0.49, *p* < 0.001) and environmental awareness (r = −0.37, *p* < 0.001), indicating that higher levels of catastrophic thinking were associated with lower problem-solving attitudes and reduced environmental awareness. Problem-solving attitudes showed a significant positive correlation with environmental awareness (r = 0.37, *p* < 0.001), suggesting that students with more positive problem-solving attitudes exhibited greater environmental awareness. The correlations among the dimensions of each construct were substantial, with catastrophic thinking dimensions intercorrelating between r = 0.60 and r = 0.69, problem-solving attitude dimensions ranging from r = 0.55 to r = 0.62, and environmental awareness dimensions correlating between r = 0.66 and r = 0.66. These moderate-to-strong intercorrelations support the multidimensional nature of each construct while confirming sufficient discriminant validity among dimensions.

To assess potential common method variance bias, Harman’s single-factor test was performed through exploratory factor analysis with all items from the three instruments loaded onto a single factor. The results indicated that the largest single factor accounted for 14.41% of the total variance, substantially below the critical threshold of 50%. This finding suggests that common method bias did not pose a significant threat to the validity of the study’s conclusions, allowing for confident interpretation of the structural relationships among variables.

**Figure 1:**
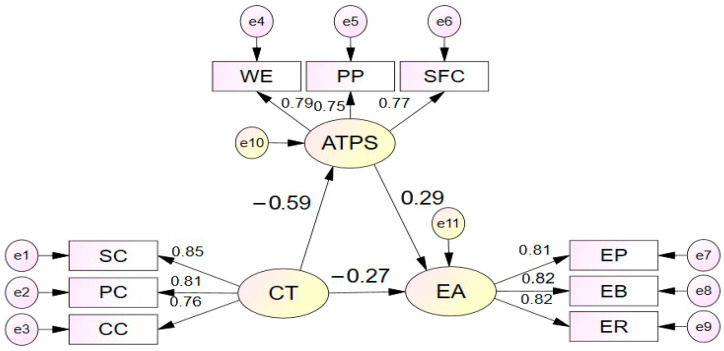
Structural Model of the Relationships Among Catastrophic Thinking, Problem-Solving Attitudes, and Environmental Awareness.

The hypothesized mediation model was tested using structural equation modeling to examine whether problem-solving attitudes mediate the relationship between catastrophic thinking and environmental awareness. The structural model, depicted in Figure 1, demonstrated excellent fit to the data across multiple indices. Specifically, the model yielded a χ^2^/df ratio of 1.037, well below the recommended threshold of 3.0, indicating minimal discrepancy between the observed and model-implied covariance matrices. The Root Mean Square Error of Approximation (RMSEA = 0.044) fell within the acceptable range, suggesting close approximate fit. Comparative fit indices further confirmed the model’s adequacy, with CFI = 0.999, TLI = 0.999, and NFI = 0.980, all substantially exceeding the conventional 0.90 criterion. Additionally, the GFI = 0.972 and AGFI = 0.966 values indicated excellent absolute fit. These convergent fit statistics provide strong empirical support for the proposed theoretical model.

The standardized path coefficients, presented in Table 3, reveal the direct effects among the latent constructs. Catastrophic thinking exerted a significant direct negative relationship with problem-solving attitudes (β = −0.592, SE = 0.062, *p* = 0.001, 95% CI [−0.700, −0.459]), indicating that higher catastrophic thinking was associated with substantially lower problem-solving attitudes. Problem-solving attitudes, in turn, showed a significant positive direct relationship with environmental awareness (β = 0.291, SE = 0.070, *p* = 0.001, 95% CI [0.164, 0.444]), indicating that more positive problem-solving attitudes were associated with greater environmental awareness. Catastrophic thinking also showed a significant direct negative relationship with environmental awareness (β = −0.266, SE = 0.083, *p* = 0.004, 95% CI [−0.415, −0.101]), indicating that catastrophic thinking was negatively associated with environmental awareness independent of its relationship through problem-solving attitudes. All factor loadings for observed indicators on their respective latent constructs were substantial and statistically significant (*p* < 0.001), with standardized loadings ranging from 0.751 to 0.848 for catastrophic thinking indicators, 0.751 to 0.789 for problem-solving attitude indicators, and 0.809 to 0.821 for environmental awareness indicators, confirming strong measurement properties.

The mediation hypothesis was examined through analysis of indirect effects using bias-corrected bootstrap confidence intervals based on 5000 resamples. As shown in Table 4, catastrophic thinking showed a significant negative indirect association with environmental awareness through problem-solving attitudes (β = −0.172, SE = 0.056, *p* = 0.001, 95% CI [−0.302, −0.080]). The bootstrap confidence interval did not contain zero, providing robust evidence that problem-solving attitudes partially mediate the relationship between catastrophic thinking and environmental awareness. This indirect pathway indicates that catastrophic thinking is associated with reduced environmental awareness partly through its negative relationship with problem-solving attitudes, which are in turn associated with environmental awareness.

The total association between catastrophic thinking and environmental awareness, which combines both direct and indirect pathways, was substantial and negative (β = −0.438, SE = 0.061, *p* = 0.001, 95% CI [−0.549, −0.309]). Comparison of the direct effect (β = −0.266) and total effect (β = −0.438) reveals that approximately 39% of the total association operates through the mediating pathway of problem-solving attitudes ([−0.172/−0.438] × 100 = 39.3%), while the remaining 61% represents the direct association between catastrophic thinking and environmental awareness. This pattern of results supports a partial mediation model, wherein problem-solving attitudes serve as a significant but not exclusive mechanism through which catastrophic thinking influences environmental awareness. The presence of both significant direct and indirect effects suggests that catastrophic thinking is associated with environmental awareness through multiple pathways: both through direct negative associations and through negative relationships with problem-solving attitudes that are linked to environmental engagement and action.

The standardized total effects presented in Table 4 further illuminate the comprehensive impact of catastrophic thinking on environmental awareness dimensions. When accounting for both direct and mediated pathways, catastrophic thinking showed negative total associations with all three environmental awareness dimensions: Environmental Pollution Awareness (β = −0.354, *p* = 0.001, 95% CI [−0.452, −0.243]), Environmental Balance Awareness (β = −0.359, *p* = 0.001, 95% CI [−0.461, −0.246]), and Environmental Resources Conservation (β = −0.360, *p* = 0.001, 95% CI [−0.458, −0.248]). These effects were remarkably consistent across dimensions, suggesting that catastrophic thinking is uniformly negatively associated with multiple facets of environmental awareness through both direct associations and indirect relationships through problem-solving attitudes. The robustness of these findings across all environmental awareness dimensions strengthens confidence in the generalizability of the mediation effect and underscores the comprehensive nature of the negative associations between catastrophic thinking and environmental cognition and concern among university students.

## 4. Discussion

The correlation analysis revealed theoretically meaningful patterns. The negative correlation between catastrophic thinking and problem-solving attitudes (r = −0.49) likely reflects how habitual worst-case scenario predictions diminish problem-solving confidence, fostering avoidance rather than approach orientations (Innocenti et al., 2023). The negative association between catastrophic thinking and environmental awareness (r = −0.37) suggests that catastrophic cognitions either deplete cognitive resources through rumination, limiting capacity for processing environmental information, or trigger defensive disengagement when overwhelming thoughts extend to environmental concerns (Pihkala, 2020; Wullenkord & Reese, 2021). The positive correlation between problem-solving attitudes and environmental awareness (r = 0.37) indicates that confidence and persistence enable sustained engagement with complex environmental issues (Schoenfeld, 1982). Notably, strong intercorrelations among catastrophic thinking dimensions (r = 0.60 to 0.69) suggest that catastrophizing operates as a generalized trait-level cognitive style rather than domain-specific worry (Petrini & Arendt-Nielsen, 2020).

The findings reveal that general catastrophic thinking shows both direct and indirect negative associations with environmental awareness among university students, with problem-solving attitudes serving as a partial mediator. The direct pathway suggests that trait-level catastrophic cognitions are independently associated with reduced environmental awareness, possibly through cognitive resource depletion and generalized avoidance tendencies that extend across life domains including environmental issues. The indirect pathway demonstrates that catastrophic thinking is negatively associated with problem-solving attitudes, which are in turn associated with environmental awareness. This dual mechanism explains approximately 39% of the total association operating through problem-solving attitudes, while 61% represents direct associations. The consistent negative effects across all environmental awareness dimensions—pollution, balance, and resource conservation—indicate that catastrophic thinking is comprehensively negatively associated with environmental cognition rather than affecting isolated aspects, highlighting its pervasive influence on ecological concern.

These results align with literature documenting that general catastrophic thinking is associated with defensive responses including avoidance and disengagement across multiple domains, particularly when self-efficacy and problem-solving confidence are compromised (Innocenti et al., 2023; Wullenkord & Reese, 2021). The negative relationship between catastrophic thinking and environmental awareness extends research on cognitive distortions by demonstrating that trait-level catastrophic tendencies—measured across social, physical, and cognitive domains—are associated with reduced awareness of environmental issues. The mediating role of problem-solving attitudes corroborates research suggesting that cognitive resources regulate the translation of awareness into effective action (Schoenfeld, 1982) and that maintaining psychological agency prevents catastrophic thinking from devolving into despair (Zheng et al., 2020). However, this study extends beyond existing cross-sectional work (Janmaimool & Khajohnmanee, 2019; Vieira et al., 2023) by integrating maladaptive cognitions and problem-solving orientations within unified analytical frameworks.

These findings carry significant implications for environmental education and mental health interventions targeting university students. Given the associations found in this study, educational programs may benefit from simultaneously addressing catastrophic cognitions while cultivating problem-solving attitudes to promote environmental awareness. Interventions might incorporate cognitive restructuring techniques to challenge worst-case scenario thinking alongside skills training in systematic problem analysis, solution generation, and persistence. Universities could implement integrated curricula combining environmental education with psychological resilience training, emphasizing actionable solutions rather than overwhelming students with apocalyptic narratives. Mental health services should recognize catastrophic thinking about environmental collapse as a legitimate concern requiring specialized support that channels ecological anxiety into constructive engagement. By fostering problem-solving attitudes, educators and counselors may help students develop the cognitive resources that are associated with higher environmental awareness rather than paralysis.

A key theoretical contribution of this study is demonstrating that general cognitive distortions—not domain-specific environmental cognitions—are associated with environmental awareness through problem-solving mechanisms. This suggests that environmental education interventions may benefit from addressing broad cognitive patterns alongside environmental content. Students exhibiting catastrophic thinking in health, social, or academic domains are associated with reduced environmental awareness through its relationship with problem-solving attitudes, even if they do not catastrophize specifically about environmental issues. This has practical implications: screening for general catastrophic thinking patterns and providing cognitive restructuring interventions may enhance environmental education effectiveness by addressing cognitive patterns that are associated with environmental engagement.

Several limitations warrant consideration when interpreting these findings. The cross-sectional design precludes causal inferences, as reverse causality remains plausible—low environmental awareness might exacerbate catastrophic thinking or undermine problem-solving confidence. The reliance on self-report measures introduces potential social desirability bias and common method variance, although Harman’s test suggested minimal concern. The sample comprised exclusively Egyptian university students from specific faculties, limiting generalizability across cultural contexts, educational systems, and age groups. Additionally, the study examined environmental awareness rather than actual pro-environmental behavior, recognizing the established awareness-behavior gap (Kollmuss & Agyeman, 2002). Unmeasured variables such as personality traits, prior environmental education, family influences, or socioeconomic factors may confound observed relationships. Future research should address these methodological constraints.

Future investigations should employ longitudinal designs to establish temporal precedence and causal directionality, examining developmental trajectories of catastrophic thinking, problem-solving attitudes, and environmental awareness throughout university education. Experimental studies testing interventions that specifically target problem-solving attitudes could determine whether enhancing these orientations effectively buffers against catastrophic thinking’s deleterious effects on environmental engagement. Cross-cultural research across diverse geographical contexts would clarify whether observed relationships generalize beyond Egyptian students. Studies should incorporate behavioral measures assessing actual pro-environmental actions rather than solely attitudinal constructs. Researchers might also explore additional mediators including self-efficacy, collective action beliefs, emotional regulation strategies, and social support systems. Investigation of moderating factors such as personality dimensions, coping styles, or exposure to environmental education would illuminate boundary conditions.

## 5. Conclusions

This study demonstrates that problem-solving attitudes are associated with both catastrophic thinking and environmental awareness, serving as a potential mediating mechanism among university students. Catastrophic cognitions are associated with reduced environmental concern both directly and indirectly through their relationships with problem-solving orientations. These findings highlight the importance of cultivating adaptive cognitive resources alongside environmental education, given the associations between catastrophic thinking patterns and reduced environmental awareness. As future professionals confronting unprecedented environmental challenges, university students may benefit from integrated support addressing both catastrophic thinking patterns and problem-solving capabilities. By fostering constructive problem-solving attitudes, educational institutions may help support environmental engagement, given the positive associations between problem-solving attitudes and environmental awareness, ultimately promoting the sustainable behaviors essential for addressing global ecological crises and achieving long-term planetary well-being.

## Figures and Tables

**Table 1:** Demographic Characteristics of Psychometric and Main Samples.

Variable	Category	Psychometric Sample		Main Sample	
		N	%	N	%
**Gender**	Male	110	16.4	304	30.7
	Female	560	83.6	685	69.3
	Total	670	100.0	989	100.0
**Academic Year**	First Year	179	26.7	248	25.1
	Second Year	140	20.9	260	26.3
	Third Year	257	38.4	226	22.9
	Fourth Year	94	14.0	255	25.8
	Total	670	100.0	989	100.0
**Residence**	Urban	180	26.9	386	39.0
	Rural	490	73.1	603	61.0
	Total	670	100.0	989	100.0

**Table 2:** Correlation Matrix of Study Variables (N = 989).

	1	2	3	4	5	6	7	8	9	10	11	12
**1. SC**	1											
**2. PC**	0.69 **	1										
**3. CC**	0.64 **	0.60 **	1									
**4. CTS**	0.90 **	0.88 **	0.82 **	1								
**5. WE**	−0.36 **	−0.35 **	−0.34 **	−0.40 **	1							
**6. PP**	−0.43 **	−0.40 **	−0.39 **	−0.46 **	0.58 **	1						
**7. SFC**	−0.36 **	−0.34 **	−0.32 **	−0.39 **	0.62 **	0.55 **	1					
**8. ATPS**	−0.44 **	−0.42 **	−0.41 **	−0.49 **	0.87 **	0.81 **	0.86 **	1				
**9. EP**	−0.26 **	−0.27 **	−0.27 **	−0.30 **	0.24 **	0.31 **	0.29 **	0.32 **	1			
**10. EB**	−0.30 **	−0.31 **	−0.28 **	−0.34 **	0.28 **	0.30 **	0.28 **	0.33 **	0.66 **	1		
**11. ER**	−0.30 **	−0.31 **	−0.27 **	−0.33 **	0.27 **	0.30 **	0.25 **	0.32 **	0.66 **	0.66 **	1	
**12. EA**	−0.32 **	−0.33 **	−0.31 **	−0.37 **	0.30 **	0.34 **	0.31 **	0.37 **	0.88 **	0.87 **	0.88 **	1

*Note.* ** *p* < 0.001.

**Table 3:** Standardized Direct Path Coefficients and Bootstrap Confidence Intervals.

Path	Estimate	SE	Lower 95% CI	Upper 95% CI	*p*
CT → ATPS	−0.592	0.062	−0.700	−0.459	0.001
CT → EA	−0.266	0.083	−0.415	−0.101	0.004
ATPS → EA	0.291	0.070	0.164	0.444	0.001
CT → CC	0.755	0.025	0.703	0.801	0.001
CT → PC	0.810	0.021	0.766	0.849	0.001
CT → SC	0.848	0.019	0.807	0.881	0.001
EB → WE	0.789	0.053	0.668	0.862	0.001
ATPS → PP	0.751	0.051	0.631	0.824	0.001
ATPS → SFC	0.765	0.057	0.624	0.841	0.001
EA → EP	0.809	0.017	0.774	0.840	0.001
EA → EB	0.818	0.017	0.783	0.851	0.001
EA → ER	0.821	0.015	0.790	0.850	0.001

*Note*. Bootstrap sample size = 5000. Bias-corrected confidence intervals.

**Table 4:** Standardized Indirect and Total Path Coefficients with Bootstrap Confidence Intervals.

Path	Indirect Effect	SE	Lower 95% CI	Upper 95% CI	*p*	Total Effect
CT → ATPS→ EA	−0.172	0.056	−0.302	−0.080	0.001	—
CT → EA (Total)	−0.438	0.061	−0.549	−0.309	0.001	−0.438

## Data Availability

The datasets generated and analyzed during the current study are available from the corresponding author upon reasonable request.

## Abbreviations

The following abbreviations are used in this manuscript:

CTS	Catastrophic Thinking Scale
SC	Social Concerns
PC	Physical Concerns
CC	Cognitive Concerns
ATPSS	Attitudes Toward Problem Solving Scale
WE	Willingness to Engage
PP	Problem-Solving Perseverance
SFC	Self-Confidence
EP	Environmental Pollution
EB	Environmental Balance
ER	Environmental Resources
EA	Environmental Awareness
CT	Catastrophic Thinking
ATPS	Attitudes Toward Problem Solving
SEM	Structural Equation Modeling
CFA	Confirmatory Factor Analysis

## Appendix A. Catastrophic Thinking Scale

Please read each statement and indicate how often you experience these thoughts using the following scale:

1 = Never | 2 = Rarely | 3 = Sometimes | 4 = Often | 5 = Always

Dimension 1: Social/Emotional Concerns

1. I fear others will think badly of me
2. I worry I’ll embarrass myself publicly
3. I think one mistake will ruin my reputation
4. I fear I’ll have an emotional breakdown in public
5. I worry others see me as incompetent
6. I believe social rejection will be devastating

Dimension 2: Physical Concerns

7. I fear my heart symptoms mean a heart attack
8. I worry bodily sensations indicate serious illness
9. I think minor pain signals a major health problem
10. I believe dizziness means I might faint
11. I fear physical symptoms are life-threatening
12. I worry unusual sensations mean something is seriously wrong

Dimension 3: Cognitive Concerns

13. I fear I’m losing control of my mind
14. I worry I might go crazy
15. I think I won’t be able to think clearly
16. I fear my mind will stop working properly
17. I worry I’m losing my mental abilities

## Appendix B. Environmental Awareness Questionnaire (Al-Dosari & Abdellatif, 2024)

Please indicate your level of agreement with each statement using the scale below:

1 = Strongly Disagree, 2 = Disagree, 3 = Neutral, 4 = Agree, 5 = Strongly Agree

Dimension 1: Environmental Pollution

1. I am aware of the major sources of air pollution in my environment.
2. I understand how water pollution affects ecosystems and human health.
3. I recognize the harmful effects of soil contamination.
4. I am knowledgeable about the impact of plastic waste on the environment.
5. I understand how noise pollution affects quality of life.

Dimension 2: Environmental Balance

6. I am aware of the importance of maintaining ecological balance.
7. I understand how human activities disrupt natural ecosystems.
8. I recognize the interconnectedness of all living organisms.
9. I am aware of how climate change affects environmental balance.
10. I understand the role of biodiversity in maintaining ecosystem stability.

Dimension 3: Environmental Resources

11. I am aware of the need to conserve water resources.
12. I understand the importance of preserving forests and natural habitats.
13. I recognize the finite nature of natural resources.
14. I am knowledgeable about renewable versus non-renewable resources.
15. I understand the importance of sustainable resource management.
